# Retraction: Chemotherapeutic Potential of 2-[Piperidinoethoxyphenyl]-3-Phenyl-2H-Benzo(b)pyran in Estrogen Receptor- Negative Breast Cancer Cells: Action via Prevention of EGFR Activation and Combined Inhibition of PI-3-K/Akt/FOXO and MEK/Erk/AP-1 Pathways

**DOI:** 10.1371/journal.pone.0231282

**Published:** 2020-03-27

**Authors:** The PLOS ONE Editors

After this article [1] was published, concerns were raised about several of the reported results.

1. In Fig 2A, there is a vertical discontinuity in the background between the NC (negative control) lane and the Control lane, and the NC lane area does not appear to contain verifiable image data. The authors commented that NC data are included in the figure and that the lack of apparent image data is due to having no background signal in this lane. The original data needed to confirm these results have not been provided.
2. Similarities were noted between the following:
  - p-NFκB and p-FOXO-3a panels in Fig 5A, especially lanes 1 and 5 of each panel: The authors stated that these are different panels.
  - MDA-MB-231 β-actin panels in Figs 5A and 6D: Authors commented that the results in the MDA-MB-231 panels of these figures are derived from the same blot, and so the same representative β -actin blot was reported in both figures.
  - p-Akt and p-NFκB panels in Fig 5B: Authors noted this image was duplicated in error, they provided an updated Fig 5B in which the p-NFκB panel is replaced.
  - Fig 6A Nuclear β-actin and Fig 6D Primary breast adenocarcinoma cells β-actin: The authors stated that these are different bands.
  - Fig 6A Cytosolic β-actin and Fig 6D Primary breast adenocarcinoma cells Rb: The authors provided an updated version of Fig 6A in which the Cytosolic β-actin panel was replaced.
  - Fig 7B cdk4, Fig 8B Cleaved PARP (horizontally flipped), and Fig 9C CTGF: The authors offered replacement panels for the Fig 7B cdk4 and Fig 9C CTGF panels, in each case noting the replacement panel is “the correct representative image”. They noted that the cleaved PARP data are correct in the published figure.
3. In Fig 9A, the right side of the Control panel appears to overlap with the left side of the EGF panel. The authors noted this was due to an error in figure preparation and they provided an updated version of Fig 9A in which the control image is replaced.
4. Fig 9C does not include a loading control blot for the western blot experiment examining CTGF levels in conditioned media. The authors noted that they did not know of an appropriate loading control for proteins in the conditioned media at the time the experiment was conducted, and that equal amounts of protein were loaded in each lane. In light of this issue, the quantification data presented in the bar graph in Fig 9C are not normalized across lanes or replicates, which raises questions about the validity of the statistical analyses.

The original data supporting the above results are not available, and so we have been unable to resolve the concerns about the published figures.

In addition, concerns were raised about the mouse tumor sizes reported in Fig 10. In response to questions about these experiments the authors commented that to prevent autoregression of tumors they initiated treatment/dosing with vehicle or compound after allowing tumors to grow for 14 days; at this timepoint the tumors had attained sizes of approximately 2000 mm^3^ as is shown in Fig 10C. They then treated the mice as described in the Materials and Methods section until animals in the control group reached 20 mm in one dimension (approximately 4000 mm^3^), at which point animals were euthanized. They also considered humane endpoints based on assessments of tumor size with respect to the percent reduction in body weight, impairment of mobility, food and water intake, and other behavioral observations. All animals in the experiment were sacrificed when the experiment was terminated on Day 30. A member of *PLOS ONE*’s Animal Research Advisory Group reviewed the article and the authors’ comments and confirmed that the tumor sizes exceed community standards for humane endpoint limits in mouse tumor studies. The advisor noted that the tumor results present a significant animal welfare and ethical concern.

In light of the above concerns, the *PLOS ONE* Editors retract this article. The editors regret that these concerns were not addressed at the time of the original review process.

AD notified the journal that the authors do not agree with retraction. RS, VC, YSP confirmed they do not agree with retraction. The other authors either could not be reached or did not respond directly.

## References

[1] Saxena R, Chandra V, Manohar M, Hajela K, Debnath U, Prabhakar YS, et al (2013) Chemotherapeutic Potential of 2-[Piperidinoethoxyphenyl]-3-Phenyl-2H-Benzo(b)pyran in Estrogen Receptor- Negative Breast Cancer Cells: Action via Prevention of EGFR Activation and Combined Inhibition of PI-3-K/Akt/FOXO and MEK/Erk/AP-1 Pathways. PLoS ONE 8(6): e66246 10.1371/journal.pone.0066246 23840429 PMC3686794

